# Intestinal Immune Imbalance is an Alarm in the Development of IBD

**DOI:** 10.1155/2023/1073984

**Published:** 2023-07-31

**Authors:** Chunli Hu, Shengtao Liao, Lin Lv, Chuanfei Li, Zhechuan Mei

**Affiliations:** Department of Gastroenterology, The Second Affiliated Hospital of Chongqing Medical University, Chongqing 400010, China

## Abstract

Immune regulation plays a crucial role in human health and disease. Inflammatory bowel disease (IBD) is a chronic relapse bowel disease with an increasing incidence worldwide. Clinical treatments for IBD are limited and inefficient. However, the pathogenesis of immune-mediated IBD remains unclear. This review describes the activation of innate and adaptive immune functions by intestinal immune cells to regulate intestinal immune balance and maintain intestinal mucosal integrity. Changes in susceptible genes, autophagy, energy metabolism, and other factors interact in a complex manner with the immune system, eventually leading to intestinal immune imbalance and the onset of IBD. These events indicate that intestinal immune imbalance is an alarm for IBD development, further opening new possibilities for the unprecedented development of immunotherapy for IBD.

## 1. Introduction

Inflammatory bowel disease (IBD) is a chronic, immune-mediated inflammatory disease characterized by the disruption of the structure and functions of the intestinal epithelial barrier [1]. IBD includes Crohn's disease (CD) and ulcerative colitis (UC), which are chronic of relapsing intestinal inflammation [2–4]. In CD, the inflammation is often transmural, whereas in UC, the inflammation is typically confined to the mucosa [5]. CD and UC differ in the presentation of some symptoms, disease location, and histopathological characteristics, but they share the manifestations of gastrointestinal symptoms such as chronic abdominal pain, intestinal obstruction or diarrhea, mucus, pus, and bloody stools, among other symptoms [6, 7]. IBD complications include strictures, abscesses, fistulas, and colitis-associated cancer [8].

The prevalence of IBD is increasing worldwide, including Europe, North America, and some developing countries [9, 10]. The prevalence of IBD is equal between men and women [11]. Several studies have reported that patients with IBD have an increased risk of colorectal cancer [12, 13] and extraintestinal malignancies (including cholangiocarcinoma, skin cancer, and hematologic malignancies) [14], which is believed to be a consequence of immunosuppressive therapies and an underlying inflammatory state [14]. There is no specific treatment method available for IBD and its related complications. Clinically, supportive treatment is the main treatment strategy [1]. The main goal of IBD treatment is to reduce the side effects during the occurrence of severe episodes, control chronic inflammation, and prevent reactivation of the intestinal inflammatory processes [15].

The etiology of IBD is complex, with a poor prognosis, and few treatment methods exist. Therefore, it is of great significance to clarify its etiology and pathogenesis to determine early diagnostic markers and effective drug targets. An increasing number of studies have revealed that IBD is caused by the interaction of multiple factors, including susceptible genes, the immune system, and host microorganisms [2, 15–17]. Among them, immune dysfunction is receiving increasing attention in the pathogenesis of IBD. Research strongly suggests that IBD is caused by disturbed mucosal immune homeostasis [7, 18, 19]. With the deterioration of the immune system, abnormal responses to the gut microbiota can trigger a series of inflammatory events that damage the intestinal wall, resulting in the disruption of the intestinal mucosal barrier and accelerating the IBD progression [20]. However, the mechanisms underlying immune-mediated IBD have not been systematically and comprehensively clarified.

**Figure 1:**
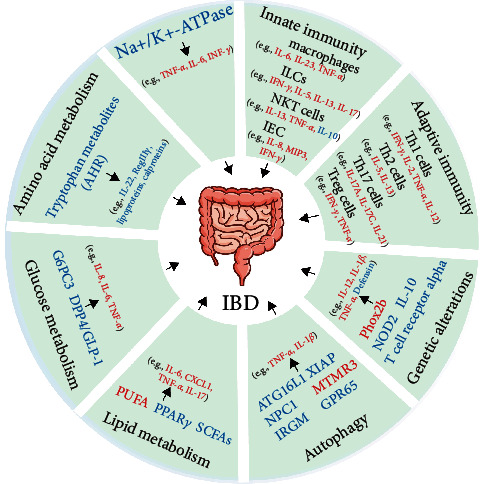
The factors of the innate immune system, adaptive immune system, genetic alterations, impaired autophagy, imbalance of energy metabolism (such as lipid metabolism, glucose metabolism, and amino acid metabolism), and electrolyte disorders interact in complex manners, which ultimately cause intestinal immune imbalance and trigger the onset of IBD. The red font indicates upregulation, while the blue font indicates downregulation. Abbreviations: ILCs, innate lymphocytes; NKT, natural killer T; IEC, intestinal epithelial cell; MIP3, macrophage inflammatory protein 3; NOD2, nucleotide-binding and oligomerization domain 2; Phox2b, paired-like homeobox 2b; Atg16l1, autophagy-related 16-like 1; XIAP, X-linked inhibitor of apoptosis; NPC1, Niemann–Pick disease type C1; MTMR3, myotubularin-related protein 3; IRGM, immunity-related GTPase M; GPR65, G-protein coupled receptor 65; PUFAs, polyunsaturated fatty acids; PPAR*γ*, peroxisome proliferator-activated receptor *γ*; SCFAs, short-chain fatty acids; G6PC3, glucose-6-phosphatase catalytic subunit 3; DPP-4, dipeptidyl peptidase 4; GLP-1, glucagon-like peptide 1; AhR, aryl hydrocarbon receptor.

Here, we reviewed the effect of immune imbalance in IBD from the perspective of immune regulation, with an attempt to provide new insights into the pathogenesis and new immunotherapeutic sites of IBD. In this review, we have provided a framework to understand the changes in the innate immune system, adaptive immune system, autophagy, gene, energy metabolism, and other factors in immune-mediated IBD, as well as to present the available research on how the abnormalities of these factors trigger immune imbalance, which may be the potential pathogenesis of experimental animal colitis and patients with IBD. Meanwhile, we have summarized the physiological and pathophysiological functions of immune cells in the intestine and discussed some of the immune sites that may act as potential therapeutic targets for IBD (Figure 1; Table 1).

## 2. Innate Immunity

### 2.1. Macrophages

#### 2.1.1. Physiological Roles of Intestinal Macrophages

In intestinal homeostasis, intestinal macrophages produce various cytokines (such as IL-10, IL-6, and iNOS) and soluble mediators (prostaglandin E2 [PGE2], bone morphogenetic protein 2 [BMP2], and WNT ligands) that are involved in the proliferation of epithelial progenitors, intestinal neuronal physiology, and endothelial physiology [21–26]. Tissue macrophages are produced by monocytes in the blood [21]. Past studies have revealed that humans possess two subsets of monocytes based on the cell surface proteins CD14^++^CD16^−^ and CD14^+^CD16^+^ [27]. Monocyte subsets in mice are divided into CX_3_CR1^int^Ly6C^hi^ and CX_3_CR1^hi^Ly6C^lo^ [28].

#### 2.1.2. Macrophage Characteristics in Mice Colitis Models and Patients with IBD

A past study reported that Ly6C^hi^ monocytes can be differentiated into CX_3_CR1^int^Ly6C^hi^ cells, which activated the Toll-like receptor (TLR) and nucleotide-binding and oligomerization domain 2 (NOD2) signal cascade components in both T cell transfer and dextran sodium sulfate (DSS)-induced mouse colitis models, reacted with bacterial products, and led to the secretion of several pro-inflammatory cytokines, including interleukin (IL)-6 and IL-23, thus promoting colitis [28].

Healthy human intestinal macrophages have no anti-inflammatory functions because they have no innate immune receptors (CD14 or TLR) [29]. Interestingly, CD14^+^ and TLR^+^ macrophage cells in the lamina propria of patients with CD increased, releasing IL-23 and tumor necrosis factor-alpha (TNF-*α*) [29, 30]; therefore, the positive innate immune receptor of myeloid cells may be closely related to IBD pathogenesis. Therefore, intestinal macrophages are considered the primary immune cells for establishing and maintaining intestinal homeostasis.

According to the description of the pathogenic role of monocytes/macrophages in experimental colitis models and patients with IBD, this further suggests that regulating monocyte/macrophage differentiation is crucial for the treatment of human IBD.

### 2.2. Innate Lymphocytes (ILCs)

#### 2.2.1. Physiological Functions of Intestinal ILCs

The ILC family includes ILC1, ILC2, and ILC3, which express lineage-specific transcription factors T-bet, GATA-3, and ROR*γ*t, respectively [31–33]. Human mucosal tissues are enriched in ILCs, and they play a key role in controlling tissue homeostasis as effector cells under the conditions of infection, immune response, and inflammation [34, 35].

#### 2.2.2. The Effects of ILCs in Mice Colitis Models and Patients with IBD

ILC1 can release interferon (IFN)-*γ* to induce inflammation in certain colitis models (Rag1^−/−^ mice treated with anti-CD40) and patients with CD [36, 37]. The depletion of intraepithelial ILC1 can ameliorate symptoms in certain colitis models (Rag1^−/−^ mice treated with anti-CD40) [36]. In addition, a past study demonstrated that IFN-*γ* produced by ILCs is an inducer of intestinal inflammation in innate colitis and that neutralization of IFN-*γ* is sufficient to ameliorate disease progression [37]. However, clinical studies have demonstrated that treatment with anti-IFN-*γ* antibody therapy did not significantly improve the clinical symptoms of patients with CD [37].

Furthermore, ILC2 in patients with UC/CD has been reported to produce IL-5 and IL-13 cytokines, which may be related to early protective immunity on mucosal surfaces [38–40]. Furthermore, IL-13^+^IFN-*γ*^+^ ILC2 was detected in intestinal samples of patients with CD, indicating that ILC2 has plastic functions under inflammatory conditions [40].

The levels of IL-17 produced by ILC3 were significantly increased in the *Helicobacter hepaticus*-induced mice colitis, the *Tbx21*^−*/*−^*Rag2*^−*/*−^ UC, and the *T-bet* knockout mice models [41–43], suggesting that ILC3 might be involved in the onset of IBD. Furthermore, IL-23 is a key cytokine driving intestinal inflammation, which is mainly related to Th17 cells [44]. ILC3 cells are the innate counterparts of Th17 cells [45]. Research has revealed that IL-23 can act on ILC3, inducing the production of IL-17 and IL-22 [46]. Consistent with this result, after IL-23 stimulation, when compared with the control group, innate immune cells isolated from patients with IBD express ILC3 genes (IL-17A, IL22, and IL23R) [44]. Studies have revealed that the expression of IL-23R on CD4^+^T cells is increased in T-cell transfer-induced colitis [47]. Therefore, IBD mouse models and patients with IBD are associated with IL23R polymorphisms, which have been reported to induce the T helper type (Th)17 cytokine (IL-17) [42, 46, 48]. Furthermore, according to genome-wide association studies (GWAS) and mouse studies, the IL23/IL-17 axis plays a pivotal role in IBD [49–53]. Therefore, ILCs play an important role in IBD pathogenesis and exhibit plasticity.

### 2.3. Natural Killer (NK) Cells

#### 2.3.1. Physiological Roles of NK and Natural Killer T (NKT) Cells

According to the cell surface density of CD56, human NK cells can be classified into CD56^dim^NK cells and CD56^bright^NK cells [54]. Past research has shown that when compared to CD56^dim^ NK cells, CD56^bright^ NK cells produced high levels of NK-derived cytokines (IFN- *γ*, IL-10, and IL-13). In the process of innate immune response in the body, the main functions of CD56^bright^ NK cells may be to provide cytokines for macrophages and other antigen-presenting cells (IFN-*γ*, IL-10, and IL-13), which effectively control infection. In addition, when compared to CD56^bright^ NK cells, CD56^dim^ NK cells are more cytotoxic [54]. NKT cells are primarily detected in the liver, thymus, and intestinal tissues' lamina propria [55, 56]. They are involved in tumor monitoring, immune response to infectious pathogens, autoimmune disease prevention, and maintenance of self-tolerance [57]. NKT cells are a unique subset of lymphocytes that express molecules from conventional T cells and NK cells [57].

#### 2.3.2. The Effects of NKT Cells in Mice Colitis Models and Patients with IBD

Studies have revealed that UC is characterized by the presence of IL-13R*α*2 NKT cells, which can be used as a marker to differentiate UC from CD [58]. DX5^+^NKT cells can play a role in downregulating the effector immune response in T-cell transfer-induced mice colitis and DSS-induced acute colitis. Mechanistically, DX5^+^NKT cells require interaction with CD1d (major histocompatibility complex class I-like molecules) to mediate their immune regulatory effects against colitis [59]. NKT cells are sources of IL-13 [60]. Compared with the healthy control group and patients with CD, IL-13 levels significantly increased in patients with UC [60, 61]. The pathogenic effects of IL-13 include the activation of inflammatory colonic mucosal NKT cells and impaired epithelial barrier function [57, 61]. Based on UC pathophysiology, activated NKT cells can strongly produce TNF-*α*-mediated immune responses [57, 60].

Based on these above-mentioned observations, the pathophysiological functions of NKT cells in experimental colitis mice models and patients with UC/CD are different. Therefore, intestinal NKT cells can secrete various cytokines to suppress (IL-10) [62, 63] or promote (IL-13) the onset of immune-mediated IBD.

### 2.4. Intestinal Epithelial Cells (IECs)

#### 2.4.1. Physiological Functions of IECs

IECs have tight junctions that defend against intestinal luminal bacteria and pathogen-associated molecular patterns, including lipopolysaccharide (LPS) [64]. In vitro studies have revealed that prolonged exposure of the intestinal epithelium to LPS-enriched conditions increases intestinal epithelial tolerance and cross-tolerance [65, 66].

#### 2.4.2. The Effects of IECs in Mice Colitis Models and Patients with IBD

Studies have shown that the mortality of TLR4 and TLR2 knockdown mice after DSS administration is higher than that of wild-type mice [65]. Furthermore, at the basal lateral of the colon epithelium, TLR5 specifically recognizes flagellin and activates nuclear factor (NF)-*κ*B, which induces the secretion of chemokines IL-8 and macrophage inflammatory protein 3 (MIP3) in model human IECs (prepared by culturing the colonic cell line T84 on collagen-coated permeable supports) [67, 68], the T cell transfer colitis model [69], and the serum of patients with CD [70].

The expression of functional TLR3 in IECs plays a crucial role in the virus-mediated innate intestinal immune response. The activation of this response causes IECs to produce IFN-stimulated gene 15 (ISG15) [70]. ISG15 (which is also known as UCRP, G1P2, IP17, IMD38, IFI15, and IMD38) is expressed at low levels in normal cells and tissues, and its expression is induced by type I IFNs (IFN-*α* and *β*) through the binding of IFN regulatory factors to IFN-stimulated response element-containing promoters [71, 72]. The mRNA expression of ISG15 was found to be increased in patients with active IBD and experimental rats with colitis, and ISG15 could enhance the IL-12-induced release of IFN-*γ* [71, 73, 74].

In short, TLRs participate in the onset of immune-mediated IBD in various ways. The interaction between damage to the IEC barrier function and an innate immune imbalance increases IBD susceptibility. Furthermore, IEC-derived cytokines enhance and prolong the inflammatory response during IBD, which is a potential immunotherapy target.

## 3. Adaptive Immunity

### 3.1. Th1 Cells

#### 3.1.1. Physiological Role of Th1 Cells in the Intestine

Type 1 immunity is particularly important for protective immunity against microbial pathogens and tumors. An uncontrolled type 1 cellular immune response leads to tissue damage, immune imbalance, and disease [75].

#### 3.1.2. Pathophysiology Th1 Cells in Mice Colitis Models and Patients with IBD

Studies have shown that during the chronic stage of DSS-induced colitis, Th1 cytokines exhibit no significant expression in the colon mucosa [76]. Earlier studies considered T cell transfer mice colitis to be a Th1-mediated model because it resulted in the development of a large population of T cells producing IFN-*γ* and TNF-*α* [77].

Studies have also reported that, compared with the control group and patients with UC, T cells in the tissues of patients with CD produce higher levels of IL-2 and IFN-*γ* [78]. In patients with CD, Th1 cell responses are associated with increased expression of IL-12 [79–81]. On the one hand, IL-12 can promote the production of IFN-*γ* by ILC1 in CD [36, 37]. On the other hand, IL-12 positively regulates IL-21 production, further facilitating the synthesis of IFN-*γ* and IL-17A [82]. Interestingly, IFN-*γ* and IL-12 can form a feedback loop. IFN-*γ* can increase macrophage-derived IL-12 production, amplifying the ongoing Th1 immune response in patients with CD [83]. IL-12 expression may be correlated with the chronic, persistent disease course of patients with CD. The CD is believed to be characterized by a Th1 immune response [20, 84–87]. Therefore, Th1 immunity is crucial in the onset of CD and is a potential therapeutic target for CD treatment.

### 3.2. Th2 Cells

#### 3.2.1. Physiological Function of Th2 Cells in the Intestine

Th2 cells are marked by higher levels of IL-5 and IL-13, along with Gata3 expression [88–90]. Type 2 immune responses promote antihelminth immunity, suppress type 1-driven autoimmune disease, and maintain metabolic homeostasis, whereas the breakdown of these mechanisms may lead to IBD [88, 91, 92].

#### 3.2.2. The Roles of Th2 Cells in Mice Colitis Models and Patients with IBD

Studies have revealed that increased levels of Th2 cytokines were noted in patients with UC and experimental colitis mice models (colitis induced by T cell transfer, 2,4,6-trinitrobenzene sulfonic acid [TNBS], and oxazolone), and the inflammation was alleviated by inhibiting Th2 cytokines [60, 61, 93–97]. UC is characterized by Th2-related cytokines, for example, IL-5 and IL-13 [60, 61, 84, 95–97].

Therefore, the pathogenesis of patients with UC and experimental colitis models is related to Th2 cell-mediated cellular immunity. This provides a new direction for UC diagnosis and immunotherapy.

### 3.3. Th17 Cells

#### 3.3.1. Physiological Functions of Th17 Cells in the Intestine

Th17 cells play important roles in maintaining symbiotic populations (We live with very large numbers [>10^14^] of microbes that have taken residence on and in ourselves, termed symbionts [98]) at important barrier sites [99]. They are a subset of T cells characterized by producing high levels of IL-17A, IL-17F, IL-21, and IL-22 [100, 101], among which IL-17A is the marker cytokine for Th17 cells.

#### 3.3.2. The Function of Th17 Cells in Mice Colitis Models and Patients with IBD

Past studies have revealed that when compared with wild-type mice, the DSS-induced mice colitis model showed significantly increased levels of IL-17C, and DSS-treated IL-17C knockout mice exhibit exacerbated intestinal inflammation [102, 103]. Furthermore, carbon monoxide can inhibit the differentiation of Th17 cells and reduce IL-17 secretion in the mouse colitis model induced by T-cell transfer and in *IL-10*-deficient mice [104].

Compared with normal intestinal mucosa, the mucosa of patients with IBD can detect high levels of IL-17A [78, 105, 106], IL-17C [102], and IL-21 [82, 107]. IL-17 plays a pathogenic role in IBD; however, certain studies have confirmed that blocking IL-17 treatment will exacerbate the symptoms of patients with moderate and severe CD [48].

Briefly, Th17 cells are indispensable in the maintenance of intestinal immune balance. Th17-related cytokines provide immunotherapeutic targets for treating patients with IBD.

### 3.4. Regulatory T (Treg) Cells

#### 3.4.1. Physiological Functions of Treg Cells in the Intestine

In the adaptive immune system, Treg cells are the key regulators of intestinal homeostasis. They secrete inhibitory cytokines IL-10 and TGF-*β*, along with the transcription factor fork box P3 (FoxP3), in the intestine primarily via the production of IL-10 to maintain intestinal mucosal homeostasis [108, 109].

#### 3.4.2. The Effect of Treg Cells in Mice Colitis Models and Patients with IBD

Studies have revealed that the adoptive transfer of Treg cells in mice colitis models induced by T cell transfer can inhibit the autoreactive T cell reaction in vivo and prevent intestinal inflammation [110, 111]. Compared with the control group, the levels of FoxP3^+^Treg cells were significantly reduced in patients with active CD, and the production of TNF-*α* and IFN-*γ* in the mucosa of patients with CD decreased after anti-TNF-*α* treatment [112]. Thus, TNF-*α* may inhibit Treg cell function in patients with active CD. However, the expressions of Treg cells are increased in patients with active UC, which is involved in inhibiting the proliferation of effector T cells and cytokine production [113]. Treg cells may differentiate into Th17 cells, resulting in IL-17^+^ and FoxP3^+^ subsets of T cells, which may explain the reduced inhibition of Treg cells in IBD [48].

Dysfunction in Treg cells is associated with the onset of immune-mediated IBD, along with effector T cells, gene mutations, and other factors. Therefore, Treg cells are crucial in maintaining intestinal homeostasis, and Treg cells are an important therapeutic target for IBD immunotherapy.

## 4. Effects of Genetic Alterations on Immune-Mediated IBD

### 4.1. NOD2

NOD2 is a putative intracellular receptor for bacterial peptidoglycans (PGNs) [114]. PGN is a polymer composed of peptide and glycan, with *N*-acetyl muramic acid and *N*-acetyl glucosamine linked via the *β*-1,4 glycosidic bond as the basic skeleton [115]. Its main function is to preserve cell integrity by withstanding turgor. PGN also contributes to the maintenance of bacterial cell shape. In addition, it is intimately involved in the processes of cell growth and cell division [116]). NOD2 is primarily expressed in monocytes, phagocytes [117, 118], and Paneth cells [114]. The NOD2 protein is involved in recognizing muramyl dipeptide (MDP), a PGN found in the cell walls of gram-positive and gram-negative bacteria [119, 120]. MDP binding activates the transcription factor NF-*κ*B, leading to the secretion of proinflammatory cytokines [120–122].

At present, several susceptibility loci (IBD1–IBD8) have been identified for CD [123], and they have the strongest association with the *CARD15* gene located within the IBD1 locus, which encodes the cytoplasmic protein [117, 118]. Caspase-activating and recruitment domain-5 (CARD5), also called NOD2, is mainly expressed in the cells of the myeloid lineage and presents with two amino-terminal CARD domains [117]. Some studies have mentioned that the *NOD2* mutation increases CD susceptibility. On the one hand, *NOD2* mutation or *NOD2*(*CARD15*) knockout mice were incapable of recognizing bacterial PGN or MDP [117, 124], increasing TLR2-mediated NF-*κ*B activation and Th1 reaction (IL-12) [117, 125]. On the other hand, studies have shown that because NOD2 and defensin are both located in Paneth cells, the mutation in NOD2 may affect the secretion of defensin in Paneth cells, but the specific underlying mechanism remains unclear [114]. In addition, it has been reported that CD-associated *NOD2* mutations result in defective gut responsiveness to LPS, possibly contributing to disease susceptibility [126, 127].

Thus, there is an antagonistic relationship between bacteria and innate immunity, which may affect the ability of the mucosa to eliminate pathogenic bacteria. The pathogenesis remains to be studied. Therefore, NOD2 acts as a protective protein in the intestine and is a potential therapeutic target for IBD treatment.

### 4.2. IL-10

IL-10 is an anti-inflammatory cytokine secreted by various immune cells (macrophages, Th17 cells, Treg cells), which can downregulate the production of Th1-derived cytokines (IL-12, IL-1*β*, and TNF-*α*) and maintain intestinal homeostasis [128–130]. Studies have reported that recombinant IL-10 administration can inhibit IL-1*β* in a concentration-dependent manner in the mucosal cultures of patients with UC [129]. Studies also revealed that continuous IL-10 administration in models of experimental colitis (*IL-10*-deficient mice, T cell transfer-induced mice colitis models, and DSS- and TNBS-induced colitis mice) can improve intestinal symptoms. Thus, endogenous IL-10 centrally regulates the mucosal immune response [128, 130]. In addition, the type of IBD diagnosed before six years of age is referred to as very early-onset IBD and presents with a strong family history [131–133]. Furthermore, IBD was diagnosed in two children with mutations in the gene encoding IL-10 [134].

Therefore, changes in the level of the *IL-10* gene play an important role in IBD development. The mouse model provides a scientific basis for patients with IBD, and the use of IL-10 as a biological agent for treating IBD requires further studies.

### 4.3. Relationship between Paired-Like Homeobox 2b (Phox2b), T Cell Receptor Alpha Gene, and IBD

GWAS have identified a single nuclear polymorphism in the enteroendocrine-associated transcription factor *Phox2b* as a risk factor for CD [135]; however, the specific underlying pathogenesis remains unclear. Studies have shown that *T cell receptor alpha* knockout mice showed significantly decreased expression of intestinal endocrine cell subpopulations (Neurotensin and Cholecystokinin [CCK] cells) and finally developed UC-like phenotypes [136]. Neurotensin regulates the colon's response to fixed stress by activating mucosal mast cells and regulating the release of colonic goblet mucin [136]. CCK regulation of intestinal growth is also related to intestinal adaptation and tumor growth [136]. Therefore, endocrine cell-related immunoregulatory molecules can maintain intestinal immune balance. However, the specific underlying mechanisms remain unclear.

## 5. The Role of Autophagy in Immune-Mediated IBD

### 5.1. Autophagy-Related 16-Like 1 (Atg16l1)

Autophagy is a mechanism for maintaining cellular homeostasis, induced by bactericidal effects and the presentation of endogenous antigens [137]. It has been reported that Ala197Thr in *Atg16l1* is associated with CD susceptibility [138, 139]. Moreover, a deficiency of *Atg16l1* in mouse spinal cord cells increased DSS-induced colitis and increased proinflammatory cytokine production (IL-1*β*, TNF-*α*) [140]. Similar studies revealed that mice with the CD-associated *Atg16l1* variant showed decreased autophagy and increased IL-1*β* production [141]. The expression of the CD-associated *Atg16l1*T300A risk allele causes defects in plasma membrane damage repair after infection with *Listeria monocytogenes* and increases cell-to-cell bacterial spread [142, 143]. Furthermore, NOD2 is involved in the autophagic response of invasive bacteria because it induces the recruitment of the autophagy protein Atg16l1 to bacterial entry sites on the plasma membrane [102, 103]; in contrast, this function is lost in cases of *NOD2* mutation [144, 145]. Thus, *Atg16l1* can maintain intestinal homeostasis and interact with various factors to affect autophagy. This further highlights the importance of Atg16l1 in IBD pathogenesis. Most research is based on animal experiments, so its role in the pathogenesis of patients with IBD remains to be elucidated.

### 5.2. X-Linked Inhibitor of Apoptosis (XIAP)

During autophagy, the inhibition or loss of XIAP function causes impaired autophagy flux, impaired bacterial clearance, and increased secretion of TNF-*α* and IL-1*β*, possibly leading to CD development [146]. XIAP interferes with NOD2-mediated cytokine production [147].

### 5.3. Niemann–Pick Disease Type C1 (NPC1)

The early onset of CD has been recently associated with NPC, a neurodegenerative lysosomal lipid storage disorder wherein the *NPC1* (*NPC intracellular cholesterol transporter 1*) gene that is involved in lipid transport is mutated [142]. Recent studies have reported that *NPC1*-related intestinal inflammation and CD-related *NOD2* and *XIAP* gene mutations cause the same functional defects, including impaired antibacterial autophagy, resulting in a similar intestinal phenotype [147]. Changes in NOD2, XIAP, and NPC1 can participate in CD development by affecting autophagy.

### 5.4. Myotubularin-Related Protein 3 (*MTMR3*)

The risk allele of *MTMR3* associated with IBD indicates increased MTMR3 levels in macrophages of IBD-risk carriers of the rs713875 CC genotype. MTMR3 can reduce the levels of pattern recognition receptor (PRR)-induced phosphatidylinositol 3-phosphate and autophagy levels, further increasing PRR-induced caspase-1 activation and autocrine IL-1*β* and NF-*κ*B signal transduction, and finally increasing the overall secretion of cytokines [141].

### 5.5. Immunity-Related GTPase M (IRGM)

IRGM belongs to the p47 immune-related GTPase family. It can resist bacterial invasion via various mechanisms, including the regulation of phagosome processing, cell motility, and autophagy [2]. Paneth cells in *IRGM*^−*/*−^ mice showed various changes, including the abnormal development of secretory granules, lowered expression of selective antimicrobial peptides (AMPs) [148], and increased susceptibility to DSS-induced colitis [148]. Furthermore, IRGM has a protective role in the intestine and participates in regulating autophagy. IRGM can maintain intestinal immune balance and can be considered a potential target for IBD therapy.

### 5.6. G-Protein Coupled Receptor 65 (*GPR65*)

Recent studies have shown that *GPR65* is a susceptible gene for IBD and an H^+^-sensitive G-protein-coupled receptor that can maintain the pH value of the lysosome, thus maintaining lysosomal energy and contributing to autophagy and pathogen defense [149]. Epithelial cells or lymphoblasts of patients with IBD and *GPR65/I231L* missense variants show abnormal lysosomal acidification, causing lysosomal dysfunction and impaired autophagy-mediated intracellular bacterial clearance [142, 149]. Compared with wild-type mice, *GPR65*-deficient mice are more likely to be infected with intestinal diseases caused by *Citrobacter*. Colitis is also more serious in these mice, along with enhanced T-cell infiltration [142]. Therefore, GPR65 is involved in the intestinal innate immune function to prevent pathogen invasion. It is therefore a potential target for IBD therapy.

Autophagy is closely related to innate immunity and is a response induced by endoplasmic reticulum stress that eventually causes apoptosis and IBD [137]. Based on genetic analyses, changes in *NOD2*, *Atg16l1*, and *XIAP* genes could lead to CD development by affecting autophagy. Therefore, the regulation of autophagy plays an important role in IBD.

## 6. Metabolic Changes Affecting Immune-Mediated IBD

### 6.1. The Role of Lipid Metabolism in Immune-Mediated IBD

#### 6.1.1. The Relationship between Polyunsaturated Fatty Acids (PUFAs) and IBD

Lipid metabolites include phospholipids, fatty acids, and cholesterol. Among these, the effects of fatty acid metabolism on immune cells have been extensively studied [150]. Food that is rich in the *ω*-6 PUFA, such as arachidonic acid (AA), increases IBD risk [151]. The possible mechanisms of *ω*-6 PUFA involvement in IBD are as follows: (i) nutrients come into direct contact with the colon mucosa, or (ii) nutrients are integrated into the colon cell membrane on the surface of the gastrointestinal tract lumen [152]. IECs from patients with CD exhibit impaired glutathione peroxidase 4 (Gpx4) activity and lipid peroxidation [151]. Furthermore, PUFAs, especially AA, induce the production of IL-6 and chemokine (C-X-C motif) ligand 1 in IECs treated with Gpx4 siRNAs in response to iron availability, lipid peroxidation, and ACSL4, similar to mechanisms of ferroptosis [151, 153]. Animal studies have revealed that mice lacking the Gpx4 allele were administered a PUFA-rich western diet, and it was found that focal granulomatous neutrophilic enteritis was triggered in IECs [151]. Changes in PUFA uptake and GPX4 activity in IECs can cause fatty acid metabolism disorders and damage epithelial barrier functions, which may be closely related to IBD development.

#### 6.1.2. Physiological Functions of Peroxisome Proliferator Activated Receptor *γ* (PPAR*γ*)

PPAR*γ* belongs to the nuclear receptor family, and it is a lipid-activated transcription factor [154, 155]. PPAR*γ* is primarily expressed in IEC in the intestine. It can be activated by fatty acids and plays a key role in regulating lipid metabolism, inflammation, cancer, insulin sensitization, and cell proliferation [155–157].

#### 6.1.3. Pathological Roles of PPAR*γ* in Immune-Mediated IBD

PPAR*γ* agonists (thiazolidinediones) inhibit the activation of NF-*κ*B through I*κ*B*α*-dependent mechanisms and further reduce cytokine expression [158]. PPAR*γ* agonists can improve inflammatory symptoms in mice colitis models, including DSS-induced mice colitis and T cell transfer-induced mice colitis models [155, 158, 159]. Interestingly, in these models, the protective effects of thiazolidinediones may be primarily mediated via hematopoietic cells because conditional PPAR*γ* deletion in the intestinal epithelium causes mild spontaneous colitis and increased susceptibility to DSS-induced colitis [160]. In addition, PPAR*γ* ligands (5-ASA) were added to the mucosal culture of patients with IBD, and they were found to increase PPAR*γ* expression [159].

Therefore, PPAR*γ* participates in regulating inflammatory reactions in the intestine and maintaining the integrity of intestinal mucosa. Although PPAR*γ* agonists are widely used in patients with IBD, the specific mechanisms underlying their anti-inflammatory effects on the colon remain unclear.

#### 6.1.4. Physiological Functions of Short-Chain Fatty Acids (SCFAs)

SCFAs include acetate, propionate, and butyrate [161] and are primarily produced by gram-negative bacteria, gram-positive bacteria, and indigestible carbohydrate fermentation [162–165]. The primary SCFA substrate is resistant starch (RS), which is an important source of butyrate [166]. In the intestinal tract, SCFA-mediated immune modulation can be regulated by various specific mechanisms: (i) activation of GPCRs, (ii) stimulation of histone acetyltransferases, (iii) inhibition of histone deacetylases (HDACs), and (iv) stabilization of hypoxia-inducible factor [167–170].

#### 6.1.5. Pathophysiology of SCFAs in Immune-Mediated IBD

In the intestinal inflammatory environment, SCFAs (including butyrate, propionate, and acetate) can bind to GPR41, GPR43, and GPR109A, activating the anti-inflammatory signaling cascade [161, 171–173]. G-protein-coupled receptors (GPCRs, GPRS) are versatile, seven-transmembrane-domain proteins that regulate a diverse array of intracellular signaling cascades [170, 174]. SCFAs are metabolites of intestinal microbiota and promote IEC RegIII*γ* in a GPR43-dependent manner along with *β*-defensins. Therefore, AMP and *β*-defensin expression were severely impaired in GPR43 knockout mice; the symptoms worsened after DSS administration, and the level of TNF-*α* and IL-17 proteins increased [175]. Similar studies revealed that inflammation was reduced after administering high-fiber food to mice models with DSS-induced colitis [161]. SCFAs can improve the symptoms of wild-type colitis mice, but the symptoms of GPR43 knockout mice did not improve [167], indicating that the initiation of SCFA anti-inflammatory mechanisms depends on GPR43.

Furthermore, butyrate induces epigenetic modifications that upregulate histone H3 acetylation of FoxP3 and induce Treg differentiation, acting as an anti-inflammatory mediator [176]. SCFAs increased the number of FoxP3^+^Treg cells in T cell transfer-induced mice model with colitis and depended on the expression of GPR43 in Tregs [176, 177].

SCFAs (including butyrate, propionate, and acetate) can recruit neutrophils, depending on GPR43. In patients with CD, the function of neutrophil recruitment is abnormal, resulting in delayed bacterial clearance and the further onset of granuloma [178]. Increased GPR43^+^ neutrophil infiltration was found in patients with CD receiving dietary fiber-supplemented (such as resistant starch, non-starch polysaccharides (e.g., celluloses, hemicelluloses, pectins, and gums), non-digestible oligosaccharides, and sugar alcohols [179]) enteral nutrition compared with patients receiving only enteral nutrition [178]. SCFA enemas increase mucosal production, crypt length, and DNA content in colon cells and improve UC symptoms in patients and rats administered with TNBS [180].

Thus, SCFAs produced via the interaction of intestinal bacteria and dietary fiber can activate innate and adaptive immune functions, maintain the intestinal barrier, and provide a new target for IBD treatment.

### 6.2. Role of Glucose Metabolism in Immune-Mediated IBD

#### 6.2.1. Glucose-6-Phosphatase Catalytic Subunit 3 (G6PC3)

Glucose is an important energy source for immune cells; these cells use glucose to generate ATP and metabolic intermediates [181]. G6PC3 is required for cellular glucose homeostasis and for catalyzing the hydrolysis of glucose 6-phosphate to glucose [182]. Interestingly, neutrophils greatly depend on glycolysis as an energy source [183–185]. Biallelic G6PC3 mutations cause the multisystem autosomal recessive disorder of G6PC3 deficiency (also known as severe congenital neutropenia type 4 [186–189]).

Individuals with severe congenital neutropenia-related IBD are deficient in G6PC3 [186, 190, 191], with CD-like inflammation, frequent stenosis, and severe oral aphthous ulcers [186, 190–193]. Previous studies have reported that G6PC3 deletion may result in the formation of IBD in the following two aspects: (1) deficiencies in the antibacterial activity of the innate immune system in patients with G6PC3-deficiency; and (2) the response of neutrophils with G6PC3 deficiency to LPSs significantly increases the levels of proinflammatory cytokines [185]. The results of similar studies are consistent with these findings. Compared with healthy controls, constitutively activated neutrophils in patients with G6PC3-deficiency released excessive amounts of soluble inflammatory mediators such as IL-8, resulting in IBD [194]. Previous studies have shown that excess IL-8 acts as a potent neutrophil chemotactic factor and activates ligands to drive tissue inflammation, which correlates with the severity of IBD [194–196]. Studies have reported that hematopoietic stem cell transplantation can be used to cure neutropenia and improve IBD caused by G6PC3 deficiency [185, 197].

Taken together, G6PC3 can maintain the homeostasis of human neutrophils. Therefore, normal glucose metabolism is extremely important for maintaining intestinal immune balance.

#### 6.2.2. Role of the Dipeptidyl Peptidase 4 (DPP-4)/Glucagon-Like Peptide 1 (GLP-1) Axis in IBD

GLPs, including GLP-1 and GLP-2, and the peptide YY are secreted by L cells, a type of enteroendocrine cell [198]. GLP-1 enhances glucose-induced insulin responses, promotes pancreatic *β*-cell survival, and exhibits anti-inflammatory properties [198]. In the present study, we mainly describe the pathophysiological functions of GLP-1 in intestinal inflammation.

Previous studies reported that GLP-1 expression was significantly downregulated in the T cell transfer-induced mice colitis model [199]. Furthermore, another study reported that excess glucose promotes the mitochondria in T cells to produce more reactive oxygen species, which activate some cytokines (TGF-*β*, IL-17, and IL-6) related to Th17 cells, leading to excessive differentiation and stimulation of Th17 cells, which triggers inflammation in vivo [200]. Therefore, GLP-1 can regulate Th17 differentiation in an inflammatory environment. However, impaired regulation of glucose metabolism caused by high glucose or GLP-1 can result in an intestinal immune imbalance.

Glucagon-like peptide 1 receptor (GLP-1R) is widely expressed in various immune cell populations, particularly in intestinal intraepithelial lymphocytes (IELs) [201, 202]. GLP-1 can bind to GPL-1R in IELs to regulate intestinal immunity. Past studies have demonstrated that the activation of GLP-1R on intestinal IEL in mice can inhibit the expression of inflammatory cytokines (such as IL-2, IL-17a, IFN-*γ*, and TNF-*α*). In DSS-induced colitis, when compared with GLP-1R^+/+^ mice, GLP-1R^−/−^ mice displayed more severe intestinal injury. Therefore, GLP-1R can improve the symptoms of DSS-induced colitis in mice [199, 201]. A similar study found that GLP-1 was significantly increased in mice with DSS-induced colitis treated with DPP-4 inhibitors [199]. Therefore, GPL-1/GPL-1R can maintain the function of the intestinal mucosal barrier.

DPP-4 (also known as CD26) is a type II integrated transmembrane glycoprotein [203]. The major substrates of DPP-4 are incretins, including GLP-1, gastric inhibitory polypeptide (GIP), which is secreted from K cells in the upper small intestine in response to the presence of nutrients, especially fats [204], and GLP-2, which is responsible for glucose metabolism [205, 206]. Apart from its roles as an enzyme, DPP-4 also plays key roles in T cell development, activation, and immune regulation [207, 208]. Functional studies on Jurkat T cell lines transfected with DPP-4 have demonstrated that the cross-linking of DPP-4 and CD3 antigens with their corresponding antibodies enhances intracellular calcium mobilization and IL-2 production, further demonstrating that DPP-4 plays an important role in T cell activation [207].

Interestingly, compared with healthy controls, serum GLP-1 levels were elevated in patients with IBD [209, 210], DPP-4 expression was increased in T cells, and DPP-4 activity was decreased in the circulation. This is considered a counter-regulatory mechanism that is conducive to limiting the occurrence of systemic immune responses [211–213]. Interestingly, DPP4 is expressed as a type II transmembrane protein. Soluble DPP4, which lacks the transmembrane and intracellular segments, is found in the circulation [214, 215].

At present, DPP-4 inhibitors are used to treat experimental mice colitis (DSS-induced mice colitis and TNBS-induced mice colitis), and their mechanism may be inhibiting IL-6 and TNF-*α*. Myeloperoxidase secretion and IL-10 upregulation ameliorate the inflammatory reaction and promote the healing of the lesions [216–219].

In summary, dysregulation of the DPP-4/GLP axis can lead to glucose metabolism disorders and immune imbalances, which further lead to IBD formation. Therefore, normal glucose metabolism can maintain the function of the intestinal mucosal barrier.

### 6.3. Role of Amino Acid Metabolism in Immune-Mediated IBD

Amino acids are essential for synthesizing various specific proteins, including cytokines and antibodies [220]. They are not only involved in the development of immune organs and the proliferation and differentiation of immune cells but also affect the secretion of cytokines and the regulation of immune responses [150]. Amino acid deficiency leads to atrophy of immune organs and dysfunction of immune cells [221].

Intestinal tryptophan (Trp) can be metabolized by the intestinal flora, Trp hydroxylase, and indoleamine 2,3-dioxygenase 1 to produce metabolites endogenous aryl hydrocarbon receptor (AhR) ligands (such as indole-3-aldehyde [IAld] and 6-formylindolo [3,2-*b*] carbazole) [222–225]. AhR is a cytoplasmic transcription factor present in many types of immune cells [226]. AhR ligands obtained from diet induce IL-22 secretion, which, in turn, facilitates intestinal production of mucins and AMP that are responsible for pathogen resistance and mucosal protection [223, 227, 228].

A study revealed that IAld activates AhR to enhance IL-22 expression in ILC3 and then stimulates epithelial cells to produce antimicrobial proteins, including REGIII*γ*, lipoproteins, and calprotectin [223]. Animal studies reported that the Th17 cell response in the intestine of AhR^−/−^ mice was significantly upregulated [223]. These studies further prove that AhR is involved in regulating the balance between ILC3 and Th17 cells and maintaining intestinal immune homeostasis.

Studies have shown that the Trp metabolism in the intestinal flora is impaired in patients with IBD; this results in AhR activation deficiency [224]. Furthermore, animal studies have reported that AhR deficiency aggravates inflammation in T cell transfer- and DSS-induced mice colitis and that it induces colitis by reducing IL-22 release [229]. Interestingly, AhR agonists and lactobacillus strains that can metabolize Trp can improve intestinal inflammation in mice [230]. These studies further indicate that AhR and AhR ligands are potential therapeutic targets for IBD.

Intestinal chromaffin cells can convert Trp into 5-hydroxytryptamine via Trp hydroxylase. There was an increase in 5-hydroxytryptamine in DSS- and TNBS-induced mouse colitis models [231, 232]. The use of a specific tryptophan hydroxylase 1 (TpH1, which is responsible for serotonin synthesis in the peripheral tissues and is mainly expressed in the enterochromaffin cells of the gut and the pineal gland [233]) enzyme inhibitor can inhibit the synthesis of 5-hydroxytryptamine and further improve the inflammatory symptoms of experimental colitis in mice [234]. In contrast, 5-HT tissue levels are significantly reduced in patients with CD and UC, and the intestinal chromaffin cells may be affected by the relevant inflammatory process [235]. However, the specific mechanism remains unelucidated.

Taken together, these results suggest that amino acid metabolites, intestinal microorganisms, and the host interact to maintain the intestinal mucosal barrier. Therefore, changes in amino acid metabolism have a potential role in IBD pathogenesis and could be new therapeutic targets for IBD.

### 6.4. Relationship between Na^+^/K^+^-ATPase and Immune-Mediated IBD

Na^+^/K^+^-ATPase is present in the basolateral membrane of villus and crypt intestinal cells. Cl^−^ secretion and Na^+^ absorption depend on the function of Na^+^/K^+^-ATPase in the basolateral membrane [236]. Therefore, Na^+^/K^+^-ATPase can maintain the electrolyte balance in the intestine.

Previous studies reported that, compared with healthy controls, the activity of Na^+^/K^+^-ATPase was decreased in patients with active UC-associated diarrhea, and its activity was found to be related to inflammatory cell infiltration [237]. Furthermore, anti-CD3mAb mice can activate T cells to produce proinflammatory cytokines, such as TNF-*α*, IFN-*γ*, and IL-6, increase mucosal permeability, and decrease Na^+^/K^+^-ATPase activity in epithelial cells, further causing transient watery diarrhea [236]. Interestingly, several studies have reported that TNF-*α* plays an important role in T cell-induced diarrhea [238–240]. Similarly, IBD-induced diarrhea is associated with enhanced T cell activation and reduced Na^+^/K^+^-ATPase activity and sodium and water reabsorption [236, 237]. In adults, approximately 8–10 l of fluid enter the intestinal cavity every day. These liquids are derived from dietary intake and intestinal contribution, and are secreted from exocrine glands. However, only 0.1–0.2 l of the liquid are excreted from the feces. Therefore, the normal gut has a strong reabsorption function [241, 242]. In addition, studies on IBD-related diarrhea have demonstrated that the colon's ability to absorb water is significantly impaired [241] and that the disruption of the intestinal barrier increases permeability, leading to an increase in water secretion [243]. In clinical treatment, TNF-*α* inhibitors, such as infliximab, have been used to prevent diarrheal symptoms in patients with IBD [244, 245]. Taken together, electrolyte balance can help maintain intestinal immune balance and protect the integrity of the intestinal mucosa.

## 7. Conclusions and Outlook

Previous studies have revealed that immune balance is important for protecting the intestinal mucosal barrier and provided convincing evidence. However, the involvement of immune imbalance in IBD pathogenesis remains completely unclear. In this field, systematic reviews highlighting the potential mechanisms in experimental mouse colitis models (T cell transfer-induced colitis, DSS-induced colitis, and TNBS-induced colitis) and patients with IBD after immune imbalance caused by multiple factors are lacking. Therefore, we hope to provide a basic and systematic review of immune regulation and IBD, which can promote researchers to pay attention to the immune regulation process from the normal intestinal mucosa to IBD. This will provide a new perspective not only for treatment but also for prevention.

## Figures and Tables

**Table 1:** Physiological function of immune cells in the intestinal tract and the pathophysiological function of inflammatory bowel disease (IBD).

Immune cell	Physiological functions in the intestinal tract	The pathophysiological role in IBD
Macrophages	Assist Treg cells' expansion and production of *IL-10* [21, 22].	Secretion of large amounts of pro-inflammatory cytokines such as IL-23 and TNF-*α* [29, 30].
ILCs	Controlling tissue homeostasis [34, 35].	ILC1 can release IFN-*γ* [36, 37, 41]; LC2 can produce IL-5 and IL-13 cytokines [40]; patients with IBD express ILC3 genes (IL17A, IL22, and IL23R) [44].
NKT cells	Response to infectious pathogens, prevention of autoimmune diseases, and maintenance of self-tolerance [57].	NKT cells are sources of IL-13 [60]. The expression of IL-13 was significantly increased in UC [60, 61].
IECs	Defend against intestinal luminal bacteria and pathogen-associated molecular patterns [64].	TLR5 recognizes flagellin, whose activation stimulates the NF-*κ*B, which induces the secretion of chemokines IL-8 and MIP3 [67, 68]. ISG15 expression was increased and ISG15 could enhance IL-12-induced IFN-*γ* release [71, 73, 74].
Th1 cells	Protective immunity against microbial pathogens and tumors [75].	In CD, Th1 cell responses are associated with an increased expression of IL-12 [79–81].
Th2 cells	Promote antihelminth immunity, suppress type 1-driven autoimmune disease and maintain metabolic homeostasis [88].	UC is characterized by Th2-related cytokines (IL-5 and IL-13) [60, 61, 95, 96].
Th17 cells	Maintaining symbiotic populations at important barrier sites [99].	The mucosa of patients with IBD can detect high levels of IL-17A [78, 105, 106], IL-17C [102], and IL-21 [82, 107].
Treg cells	Tregs act as the key regulators of intestinal homeostasis [108, 109].	Tregs may differentiate into Th17 cells, resulting in the IL-17^+^ and FoxP3^+^ subsets of T cells [48].

IL, interleukin; TNF-*α*, tumor necrosis factor-alpha; ILC, innate lymphocyte; IFN, interferon; NKT, natural killer T; IEC, intestinal epithelial cell; UC, ulcerative colitis; TLR, Toll-like receptor; NF, nuclear factor; ISG15, IFN-stimulated gene 15; MIP3, macrophage inflammatory protein 3; CD, Crohn's disease; Treg, regulatory T.

## Data Availability

The data are available from the corresponding author upon reasonable request.
